# Cytoreductive Nephrectomy and Nephrectomy/Complete Metastasectomy for Metastatic Renal Cancer

**DOI:** 10.1100/tsw.2007.145

**Published:** 2007-02-19

**Authors:** Paul Russo, Mark Synder, Andrew Vickers, Varuni Kondagunta, Robert Motzer

**Affiliations:** Departments of Surgery, Urology Service (P.R., M.S.), Medicine (R.M., V.K.) and Epidemiology and Biostatistics (A.V.), Memorial Sloan Kettering Cancer Center, 1275 York Avenue, New York, NY 10021, USA

**Keywords:** renal cancer, cytoreductive nephrectomy, metastasectomy

## Abstract

The objective of this study was to determine our institutional experience with cytoreductive nephrectomy alone or in conjunction with nephrectomy complete metastasectomy. Between July 1989 and September 2003, we queried our department's renal tumor database for patients undergoing cytoreductive nephrectomy alone or in conjunction with complete metastasectomy. Clinical and pathological factors analyzed included primary tumor size, stage and histological subtype, age, gender, Karnofsky Performance Status (KPS) prior to nephrectomy, number and location of metastatic sites, and the presence or absence of any systemic therapy. Preoperative laboratory values analyzed included hemoglobin (HGB), calcium (CA), albumin (ALB), lactose dehydrogenase (LDH), alkaline phosphatase (ALP), and corrected calcium. Corrected calcium was defined as follows: corrected calcium = total calcium - 0.707*(albumin - 3.4). During this time frame, 1628 patients underwent nephrectomy (partial or radical) for renal masses, 91 (5.6%) of whom had metastatic disease. In this group, 71% of patients were male, 88% of patients had a KPS of 80% or greater, and 92% had conventional clear cell histology. Sixty-four percent of patients had a single site of metastatic disease, with lung the most common, followed by bone, adrenal, brain, and liver. Sixty-one patients (67%) had nephrectomy with removal of all metastatic sites (nephrectomy/complete metastasectomy) and 30 (33%) had cytoreductive nephrectomy alone. Median survival for patients undergoing nephrectomy/complete metastasectomy was 30 months. Median survival for patients undergoing cytoreductive nephrectomy alone was 12 months. Perioperative complications occurred in 13% of patients and four patients died within 30 days of their operation. For patients with metastatic renal cell carcinoma, surgical resection of the primary tumor alone (cytoreductive nephrectomy) or in conjunction with metastasectomy can be accomplished with acceptable perioperative morbidity and mortality. This surgical experience provides a contemporary foundation as new targeted therapeutic agents are integrated into the neoadjuvant or adjuvant treatment of locally advanced and metastatic renal cancer.

## INTRODUCTION

In the year 2006, there will be an estimated 38,890 new cases and 12,840 deaths from kidney cancer in the U.S.[1]. Since 1950, there has been a 126% increase in the incidence of renal cancer and a 36.5% increase in annual mortality[2]. Two general groups of patients with renal cortical tumors (RCT) exist: those (approximately 70% of the total) with small (T1, median tumor size 4.0 cm), incidentally discovered RCT with an excellent prognosis (>90% 5-year survival) and those with large, symptomatic, locally advanced tumors that either present with or later become metastatic (30%) and have a guarded or poor prognosis (<10% 5-year survival). In the last decade, our understanding of RCT has evolved and it is now understood that these are a family of neoplasms with distinct histopathological appearances, unique cytogenetic defects, and variable metastatic potential ranging from the benign renal oncocytoma, to the indolent papillary and chromophobe carcinomas, to the conventional clear cell carcinoma that accounts for 65% of the RCT, but 90% of those that metastasize[3].

Operative management of small RCT today consists of open or laparoscopic partial nephrectomy and radical nephrectomy when partial nephrectomy is not technically feasible, to achieve the concomitant goals of local tumor control and maintenance of maximal renal function[4,5]. For massive, locally extensive renal tumors with or without involvement of the renal vein, inferior vena cava, and regional nodes, radical nephrectomy with removal of perinephric soft tissues, ipsilateral adrenal, and regional lymph nodes is indicated[6]. Historically, for the patients who present with or later developed metastatic disease, systemic therapies (including systemic chemotherapy, hormonal therapy, and cytokines alone or in combination) were generally ineffective with overall response rates that rarely exceeded 20% and median survival rates of 10–13 months[7]. Survival for newly diagnosed or previously treated metastatic patients is directly related to selection factors as described by Motzer and colleagues at Memorial Sloan Kettering Cancer Center (MSKCC)[8,9]; adverse prognostic factors for a shorter survival, low performance status (Karnofsky performance status, KPS, <80% or ECOG performance status >1), high LDH (>1.5 × upper limit of normal), low hemoglobin, high corrected serum calcium (>10 mg/dl), and the absence of nephrectomy[8,9].

The role of surgical resection of the renal tumor primary and synchronous or asynchronous metastatic sites (*nephrectomy complete metastasectomy*), or the resection of the renal tumor primary alone in the face of unresectable metastatic disease (*cytoreductive nephrectomy*), has long been controversial with data insufficient to determine if survival following operation was a therapeutic effect or simply related to patient selection factors and disease natural history. It was the purpose of the present report to review the rationale for surgical intervention in the face of metastatic disease as well as to report a contemporary experience at our center with both procedures relative to survival and perioperative complications.

## PATIENTS AND METHODS AND MATERIALS

### Patients

After receiving approval from our Institutional Review Board, we queried our departmental renal tumor surgical database, which has been prospectively updated since its inception in 1989, for patients who underwent a nephrectomy in the face of metastatic disease between July 1989 and September 2003. The database was also queried to identify patients who underwent a complete metastasectomy prior to, during, or subsequent to the nephrectomy. *Nephrectomy/complete metastasectomy* were defined as the surgical resection of all clinically evident metastatic disease sites and the tumor-bearing kidney. Patients with unresectable metastatic disease or those patients in whom an incomplete or palliative resection of metastatic sites was performed were classified as having undergone a *cytoreductive nephrectomy*.

Clinical and pathological factors analyzed included primary tumor size, stage and histological subtype, age, gender, KPS prior to nephrectomy, number and location of metastatic sites, and the presence or absence of any systemic therapy. KPS was taken from prenephrectomy evaluations or assigned retrospectively by one of us (PR) for 37 patients who were missing that information. Multiple metastatic deposits in one location were classified as involvement of a single site. For example, bilateral lung nodules counted as one metastatic site location, the lungs. Preoperative laboratory values analyzed included hemoglobin (HGB), calcium (CA), albumin (ALB), lactose dehydrogenase (LDH), alkaline phosphatase (ALP), and corrected calcium. Corrected calcium was defined as follows: corrected calcium = total calcium - 0.707*(albumin - 3.4).

### Statistical Analysis

Overall survival for patients who underwent a cytoreductive nephrectomy with complete metastasectomy and cytoreductive nephrectomy alone (including cytoreductive nephrectomy plus incomplete or palliative metastasectomy) were determined. The endpoint in these studies was death from any cause, with survival probabilities estimated by Kaplan-Meier methods[10]. Analyses were conducted using Stata 9.2 (Stata Corp., College Station, TX.).

## RESULTS

Between July 1989 and September 2003, 1628 patients underwent nephrectomy (partial or radical) for renal cortical masses. The focus of this study was the 91 (5.6%) patients with metastatic disease treated in this time frame (Table 1). Surgical characteristics are given in Table 2. Sixty-one patients (67%) had removal of the kidney and all metastatic sites (nephrectomy/complete metastasectomy), and the remaining 30 (33%) had cytoreductive nephrectomy alone without resection of all metastatic sites. There were 13 perioperative complications (14%) including five pulmonary (PE, pneumonia, respiratory failure, mucous plugging, pneumothorax), three infectious (UTI, line sepsis, wound infection) and two cardiac (bundle branch block, supraventricular tachycardia). Four patients (4%) died within 30 days of operation from disease progression.

Patient and surgical characteristics for the patients undergoing nephrectomy/complete metastasectomy and cytoreductive nephrectomy alone are given in Table 3. The groups were similar for all variables other than, as expected, the number of metastatic sites: 44 (72%) of metastasectomy patients had a single site and the remaining 17 (28%) had two sites; the number of patients with one, two, and more than two metastatic sites in patients undergoing cytoreductive nephrectomy was 14 (47%), 12 (40%), and 4 (13%).

There were 74 deaths. Median follow-up for survivors was 43 months. Median survival for patients undergoing nephrectomy/metastasectomy was 30 months (Fig. 1). Median survival for patients undergoing cytoreductive nephrectomy was 12 months (Fig. 2).

## DISCUSSION

The role of operative intervention in patients with metastatic renal cancer has long been controversial. In 1939, Barney and Churchill first reported a patient that underwent both nephrectomy for a renal cancer and excision of a solitary pulmonary metastasis only to die 23 years later of coronary artery disease[11]. Through the last 60 years, the surgical resection of limited metastatic disease (metastasectomy) was the only therapeutic alternative for such patients due to a lack of effective systemic therapies. The reported selection criteria for this aggressive approach varied from study to study, and significant prognostic factors reported included the site and number of metastatic deposits, completeness of resection, patient performance status, and the disease-free interval from nephrectomy to the diagnosis of metastatic disease. Complete resection of isolated metastases was associated with a 5-year survival rates reported between

35–60%. Despite successful resection of metastatic disease and associated patient survival, evidence was lacking that the surgical intervention itself, as opposed to patient selection factors and the natural history of the renal cancer, lead to the observed outcome[12,13,14,15].

**TABLE 1**
**Patient and Tumor Characteristics of the Study Sample***

| Patient Demographics | n = 91 |
|---|---|
| Age (years) | 61 (52, 68) |
| Gender | |
| Male | 65 (71%) |
| Female | 26 (29%) |
| Preoperative KPS | |
| ≥90 | 50 (55%) |
| 80 | 30 (33%) |
| ≤70 | 11 (12%) |
| Preop labs | |
| Calcium (mg/dl) | 9.4 (9.2, 9.9) [6] |
| Albumin (g/dl) | 4.2 (3.9, 4.4) [6] |
| Hemoglobin (g/dl) | 12.5 (10.9, 13.9) [2] |
| LDH (Units/L) | 159 (142, 186) [6] |
| ALP (Units/L) | 84 (64, 102) [16] |
| Corrected calcium (mg/dl) | 9.0 (8.6, 9.3) [8] |
| Primary tumor size (cm) | 7.9 (5, 10.5) |
| Nephrectomy type | |
| Laparoscopic radical | 5 (5%) |
| Open partial | 8 (9%) |
| Open radical | 78 (86%) |
| Bilateral nephrectomies | 7 (8%) |
| Histologic subtype | |
| Conventional (clear cell) | 84 (92%) |
| Papillary | 2 (2%) |
| Collecting duct | 1 (1%) |
| Unclassified | 1 (1%) |
| Multiple subtypes | 3 (3%) |
| Number of metastatic sites at nephrectomy | |
| 1 | 58 (64%) |
| 2 | 29 (32%) |
| >2 | 4 (4%) |
| Primary metastatic sites | |
| Lung | 44 |
| Bone | 32 |
| Adrenal | 19 |
| Brain | 10 |
| Liver | 10 |
| Pancreas | 4 |
| Other | 8 |
| Type of surgery | |
| Cytoreductive nephrectomy alone | 30 (33%) |
| Cytoreductive and metastasectomy | 61 (67%) |

| | |
|---|---|
| Perioperative complications | 12 (13%) |
| 30-day mortality | 4 (4%) |

* Values are number (%) or median (interquartile range). Figures in square brackets give the number of missing patients.

**TABLE 2**
**Surgical Characteristics**

| | |
|---|---|
| Nephrectomy type | |
| Laparoscopic radical | 5 (5%) |
| Open partial | 8 (9%) |
| Open radical | 78 (86%) |
| Bilateral nephrectomies | 7 (8%) |
| Number of metastatic sites at nephrectomy | |
| 1 | 58 (64%) |
| 2 | 29 (32%) |
| >2 | 4 (4%) |
| Primary metastatic sites | |
| Lung | 44 |
| Bone | 32 |
| Adrenal | 19 |
| Brain | 10 |
| Liver | 10 |
| Pancreas | 4 |
| Other | 8 |
| Type of surgery | |
| Cytoreductive nephrectomy alone | 30 (33%) |
| Cytoreductive and metastasectomy | 61 (67%) |

Recent metastasectomy studies also identified favorable clinical and surgical characteristics associated with an enhanced outcome, but metastasectomy has never been addressed in a large, randomized, prospective clinical trial. In 1992, Pogrebniak and colleagues[16] reported 23 patients who underwent resection of pulmonary metastases from RCC, 15 of whom had previously been treated with IL-2–based immunotherapy. Patients with resectable lesions had a longer survival (mean 49 months) than those patients with unresectable lesions (mean 16 months). In this study, too, survival was not dependent on the number of nodules removed[16]. The authors concluded that patients with metastatic RCC should be offered an operation if it is likely to be a curative metastasectomy. Favorable subgroups include those patients with a solitary site of metastases and disease-free interval of greater than 1 year. In a report from MSKCC in 1998, prognostic factors associated with enhanced survival in 278 patients who underwent surgical metastasectomy included a disease-free interval of greater than 12 months (55 vs. 9% 5-year overall survival), solitary vs. multiple sites of metastases (54 vs. 29% 5-year overall survival), and age younger than 60 years (49 vs. 35% 5-year survival). The survival was longer when the solitary site of resection was lung (54% 5-year survival) compared to brain (18% 5-year survival). Twenty-nine percent of patients with completely resected multiple sites of metastases within a given organ survived 5 years; again suggesting that complete resection of all metastatic deposits within an organ was more important than the number metastatic deposits within the organ[17]. Although the curative impact of metastasectomy is not certain, operative intervention can be indicated to provide effective palliation for symptomatic metastatic disease to sites such as bone, brain, and adrenal gland[18,19].

The role of radical nephrectomy in patients with extensive metastatic renal cancer where complete metastasectomy is not possible is also controversial. The rationale for cytoreductive radical nephrectomy relates to the theoretical purpose of removing a large, potentially immunosuppressive tumor burden that could serve as a potential source for tumor-related factors that facilitate the metastatic process, enhance tumor neovascularity, and allow tumor cells to escape immune recognition. Rarely, cytoreductive nephrectomy is performed to treat complications from the primary tumor during systemic therapy, such as unremitting gross hematuria or flank pain not relieved by conservative measures, such as angioinfarction.

**TABLE 3**
**Clinical Characteristics of Patients by Operation***

| | Complete Metastasectomy (n = 61) | Incomplete or No Metastasectomy (n = 30) |
|---|---|---|
| Age (years), median (range) | 60 (50, 66) | 63 (52, 69) |
| Gender: # (%) | | |
| Male | 47 (77%) | 18 (60%) |
| Female | 14 (23%) | 12 (40%) |
| Tumor size (cm), median (25th, 75th percentile) | 7.1 (4.8, 10.8) | 8.0 (5.9, 10.3) |
| Preop labs | | |
| Hemoglobin | 12.6 (11.8, 14.3) [2] | 11.7 (9.8, 13.3) [0] |
| Calcium | 9.5 (9.2, 10.1) [6] | 9.4 (9.1, 9.9) [0] |
| Albumin | 4.3 (4.2, 4.3) [5] | 4.2 (3.7, 4.4) [1] |
| LDH | 160 (145, 185) [6] | 155 (141, 193) [0] |
| ALP | 85 (61, 100) [14] | 78 (66, 122) [2] |
| Corrected calcium | 9.0 (8.6, 9.5) [7] | 8.9 (8.6, 9.3) [1] |
| KPS # (%) | | |
| ≥90 | 36 (59%) | 14 (47%) |
| 80 | 18 (30%) | 11 (37% |
| ≤70 | 7 (11%) | 5 (17%) |
| Histology | | |
| CLR | 55 (90%) | 29 (97%) |
| PAP | 2 (3%) | - |
| Other** | 4 (7%) | 1 (3%) |
| pT stage | | |
| pT1 | 15 (23%) | 5 (17%) |
| pT2 | 8 (13%) | 6 (20%) |
| pT3a | 24 (39%) | 8 (27%) |
| pT3b,c, pT4 | 14 (23%) | 11 (37%) |
| Number metastatic sites | | |
| 1 | 44 (72%) | 14 (47%) |
| 2 | 17 (28%) | 12 (40%) |
| >2 | 0 | 4 (13%) |
| Metastatic location | | |
| Lung only | 13 (21%) | 11 (37%) |

* Values are number (%) or median (interquartile range). Figures in square brackets give the number of missing patients.

** Other histological subtypes include: unclassified (# = 1), collecting duct (# = 1), and multiple tumors of discordant histological subtypes in one kidney (# = 3).

Radical nephrectomy should not be done in order to induce spontaneous remission, a phenomena observed only in 4/474 patients (0.8%) so treated in an important study reported from the Cleveland Clinic[20]. Surgical mortality has been reported between 2–11% for patients with large primary renal tumors and metastatic disease. Not all patients undergoing cytoreductive nephrectomy benefit from the operation. In a 1997 report from the National Cancer Institute, of 195 patients with metastatic RCC, 121 (62%) were eligible for high-dose IL-2 following cytoreductive nephrectomy leading to a reported response rate of 18%. In that series, 38% of the patients who underwent nephrectomy ultimately did not receive any immunotherapy either because of complications of the nephrectomy or rapid deterioration from disease progression[21]. Some oncology groups recommended adjuvant radical nephrectomy only if

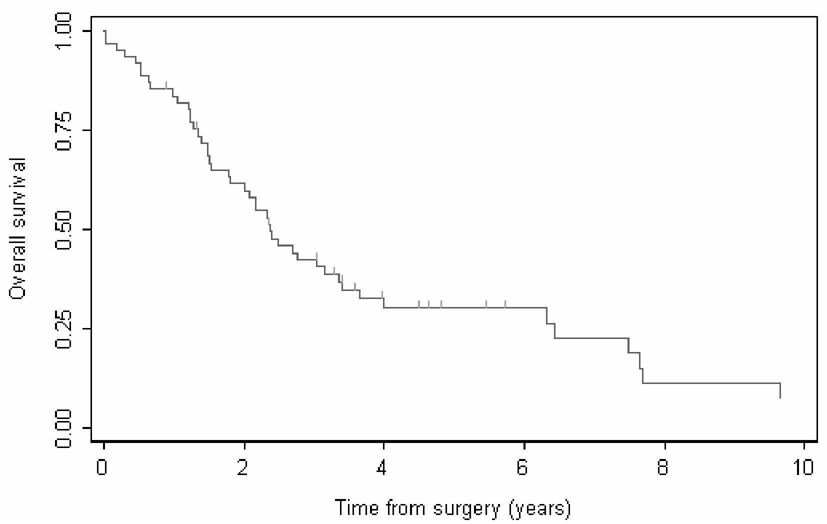

**FIGURE 1**. Survival distribution: nephrectomy plus metastasectomy.

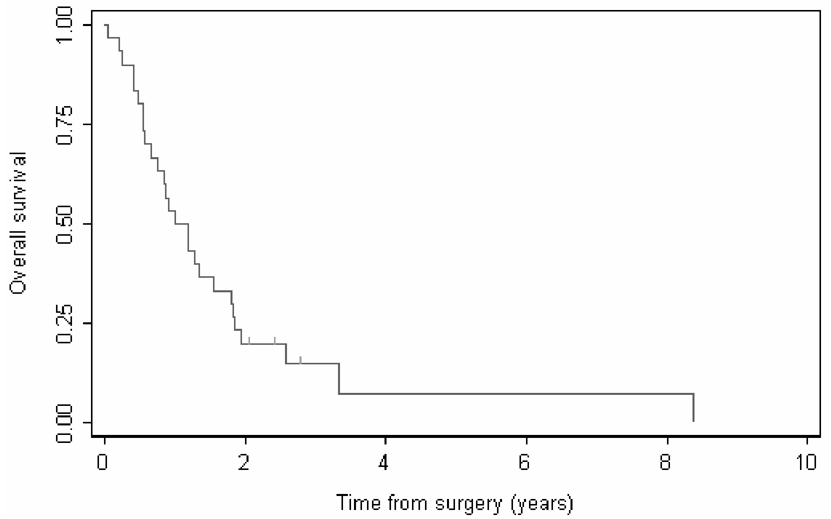

**FIGURE 2.** Survival distribution: cytoreductive nephrectomy alone.

initial systemic therapy was effective in initiating clinical regression of metastatic sites and thus avoid the surgical morbidity[22,23].

In an attempt to address this important clinical issue of the role of nephrectomy in patients with widely metastatic renal cancer, in 2001, two randomized and prospective clinical trials were completed in the U.S. (Southwest Oncology Group, SWOG, 246 patients)[24] and Europe (European Organization Research and Treatment of Cancer, EROTC, 85 patients)[25]. Both studies utilized similar entry criteria and compared cytoreductive nephrectomy plus interferon alpha 2b vs. interferon alpha 2b alone. In the SWOG trial, patients who underwent cytoreductive nephrectomy interferon alfa 2b had a significantly improved median survival of 11.1 months compared to 8.1 months for those treated with interferon alfa 2b alone[24]. In the EROTC trial, the survival advantage for cytoreductive nephrectomy plus interferon alpha 2b (17 months) was significantly greater than interferon alpha 2b alone (7 months). When the investigators from these two organizations combined their data to analyze a total of 331 patients, a median survival of 13.6 months for the cytoreductive nephrectomy plus interferon arm vs.7.8 months for interferon alone arm was reported[26]. In both series, the effect of systemic interferon was negligible. Unlike historical series, operative mortality in the combined experience was 1.5% (two patients) and only 5.6% of patients did not proceed to interferon therapy after nephrectomy, which may be a result of improved surgical skills and perioperative care compared to historical series. Experienced laparoscopic surgeons have performed cytoreductive nephrectomy in carefully selected patients and reported decreased blood loss, less analgesic support, shorter hospitalization, and sooner initiation of systemic therapies[27]. Careful patient selection for any approach to cytoreductive nephrectomy is required with special attention to regional nodal encasement of the hilar vessels, tumor neovascularity, inferior vena caval extension, and the general health of the patient.

Despite the apparent survival benefit of the cytoreductive nephrectomy as described above, the impact of referral patterns, surgical judgment, and patient selection was reported by Bromwich and colleagues[27] from Glasgow, U.K. In their study, 94 patients with metastatic renal cancer were evaluated between 1998–2001 for possible cytoreductive nephrectomy. Of these patients, 38 (40%) were considered inoperable by surgical staff and 36 patients were felt to have an ECOG performance status of >1. Cytoreductive radical nephrectomy was offered to 20 patients (22%) with a performance status of 0 or 1 and performed in 19. Of the 19, 13 patients began a course of immunotherapy (interferon) after operation. Seven patients had treatment-related toxicity necessitating withdrawal from the study and four patients had progressive cancer despite the interferon. Four patients were alive after cytoreductive nephrectomy (mean 8 months, range 3–16 months) and 15 have died of disease (mean time to death 9.5 months, range 3–28 months)[28]. The general impact of cytoreductive surgery and systemic therapy in this group of patients was minimal.

The extent to which the patient selection factors described by Motzer and colleagues at MSKCC[8,9] (KPS, serum corrected calcium >10 mg/dl, hemoglobin less than the sex-specific lower limit of normal, and LDH >1.5× the upper limit of normal) and validated externally[29] could apply to a population of initially nonmetastatic surgical patients who later developed metastatic renal cancer was recently reported by MSKCC investigators[30]. A group of 118 patients, 79% with conventional clear cell carcinoma and 60% with tumors of pathological T3 or higher, were stratified according to the above risk factors. Time from nephrectomy to recurrence was <12 months in 50 patients (42%, median 4 months, range 1–11 months) and >12 months in 68 patients (58%, median time from nephrectomy to recurrence 32 months range 12–136 months). An inverse correlation between risk factors and survival existed. Median survival for low-risk patients (34% of total, zero risk factors) was 76 months, intermediate risk (50% of total, one or two risk factors) was 25 months, and for high-risk patients (16% of total, three to five risk factors) was 6 months. Thirty patients (25%) in this dataset ultimately underwent surgical metastasectomy, of whom 46% were in the low-risk group. The risk stratification described above has important implications for adjuvant therapy trials, particularly in the low-risk patients whose natural history could indicate a relatively long survival with or without systemic treatment. These data also suggest that the relative wellness of the patient (absence of risk factors) was an important part of the surgeon’s decision to perform metastasectomy.

For over 30 years, extensive clinical research with systemic cytokines, chemotherapeutic agents, hormonal manipulations, and vaccines have been largely unsuccessful in treating metastatic renal cancer, leading many investigators to conclude that this disease is resistant to systemic therapy[7]. However, targeted therapeutic approaches to RCC have recently become available with the understanding of the molecular events associated with the germline inactivation of the von Hippel-Lindau (VHL) tumor suppressor gene responsible for this hereditary cancer syndrome, and somatic mutations of this gene that lead to the development of sporadic hemangioblastoma and conventional clear cell carcinoma of the kidney. The VHL gene produces a protein (pVHL) that works through its oxygen-dependent polyubiquitylation of HIF (hypoxia-inducible factor), which is critical in mammalian oxygen sensing apparatus. In the absence of normal pVHL, HIF is stabilized and induces the expression of its target genes that are critical in tumor cell induced angiogenesis, cell growth, and cell survival[31]. The elucidation of this cellular pathway lead to robust preclinical[32] and clinical studies using monoclonal antibodies to bind and inactivate vascular endothelial growth factor (VEG-F), tyrosine kinase inhibitors that block the receptors for VEG-F and platelet derived growth factor (PDF-F), and mammalian target of rapomycin inhibitors (mTOR), a serine/threonine kinase responsible for numerous cell maintenance and cell cycle functions[33,34,35,36,37].

Two oral multitargeted kinase inhibitors, sorafenib and sunitinib, have undergone extensive clinical trials in previously cytokine-treated patients with metastatic renal cancer as second-line therapy and have been effective in this setting of inducing partial remissions, disease stabilization, and delay in time to progression of up to 8.7 months in patients previously without a therapeutic option. Of interest is the observation in some cases of substantial regression of the primary kidney tumor during targeted therapy. Regression of primary and metastatic sites has been associated with qualitative changes in imaging contrast uptake, suggesting an effect on the vascular perfusion by the systemic agents[38,39]. In a multi-institutional, open-label, single-arm clinical trial of sunitinib in 105 patients who previously failed cytokine therapy, the objective response rate was 34% partial response rate and a median progression free survival of 8.3 months[40]. The most common adverse effects of these agents are generally manageable in under 30% of patients and include fatigue (28%), diarrhea, hand and foot syndrome, rash, and hair loss when the rash involved the scalp.

In our dataset, which spanned a 14-year period, operations in the face of metastatic renal cancer represented a small percentage of the overall surgical experience (<6%) at our center. The 61 patients that underwent a nephrectomy and metastasectomy differed from the 30 patients undergoing cytoreductive nephrectomy by only the preoperative selection factor of serum hemoglobin (12.6 vs. 11.7g/dl). However, the more limited extent of disease that allowed for these carefully selected patients to undergo metastasectomy likely explains their enhanced survival compared to patients undergoing cytoreductive nephrectomy in which metastasectomy was not possible (30 vs. 12 months).

For the entire group, perioperative complications were acceptable (13%), but four patients died of disease progression within 30 days. We cannot necessarily ascribe the observed differences in outcome to differences in surgical approach. Selection bias may take place preoperatively, in terms of a surgeon’s or patient's willingness to undergo a potentially complex radical nephrectomy when overall anticipated survival is limited. Perhaps more critically, selection bias acts intraoperatively, as patients seen to have widespread or unresectable disease will receive only cytoreductive nephrectomy.

As new and possibly more effective systemic agents are introduced for the treatment of metastatic renal cancer, shifting surgical strategies may be required. Future clinical trial design for patients with widely metastatic renal cancer will likely integrate pretreatment with effective systemic targeted agents with surgical intervention as a means of consolidation, either in the form of cytoreductive nephrectomy and/or metastasectomy, followed with the possibility for long-term maintenance of the systemic agents. Surgical debulking of the primary and, if possible, complete metastasectomy, coupled with effective systemic targeted agents, may convert metastatic renal cancer from a progressive disease to a chronic disease. Our study provides a baseline of expectations and perioperative complications in a contemporary series of patients undergoing operative resection in the face of metastatic renal cancer.

## CONCLUSIONS

Patients with RCC that either present with or later develop metastatic disease represent approximately 30% of the patients with RCT. Operative intervention in patients with metastatic renal cancer has long been controversial and subjected to extensive selection biases. Our 14-year experience with 91 patients undergoing operative intervention either as a nephrectomy/metastasectomy (n = 61) or as a cytoreductive nephrectomy (n = 30) is presented. Survival differences, median of 30 months in the former group and 12 months in the latter, are likely associated with the extent of disease at the time of intervention and patient selection factors. As new, recently FDA-approved targeted agents, such as sunitinib and sorafenib, with specific activity in metastatic renal cancer, are integrated into the treatment of locally advanced and metastatic renal cancer, the role of the surgical intervention is likely to evolve with cytoreductive nephrectomy and metastasectomy will be utilized to achieve complete surgical remission. Our data also indicate that operative intervention in the face of metastatic renal cancer can be done with acceptable surgical morbidity.

## ACKNOWLEDGMENT

Presented at the Annual Meeting of the Society of Surgical Oncology, Atlanta Georgia, March 2005.

## References

1. Jemal, A., Siegel, R., Ward, E., et al. (2006) Cancer statistics, 2006. *CA Cancer J. Clin.* 56: 106–130.
2. Pantuck, A.J., Zisman, A., and Belldegrun, A.S. (2001) The changing natural history of renal cell carcinoma. *J. Urol.* **166,** 1611–1623.
3. Reuter, V.E., Russo, P., and Motzer, R.J. Renal cancer pathology and prognostic factors. In: Vogelzang, Nicholas J. Genitourinary Oncology. 3rd ed. Philadelphia: Lippincott Williams and Wilkins; 2006. p. 681-697.
4. Russo, P. (2006) Renal tumors: developing understanding leads to developments in surgical treatment. *BJU Int*. **97,** 9–10.
5. Russo, P. (2006) Open partial nephrectomy: an essential contemporary operation. *Nat. Clin. Pract. Urol.* **3,** 2–3.
6. Russo, P. (2006) Open radical nephrectomy for localized renal cell carcinoma. In *Genitourinary Oncology.* 3rd ed. Lippincott Williams and Wilkins, Philadelphia, PA. pp. 725–731.
7. Motzer, R.J. and Russo, P. (2000) Systemic therapy for renal cell carcinoma. *J. Urol.* **163,** 408–417.
8. Motzer, R.J., Mazumdar, M., Bacik, J., et al. (1999) Survival and prognostic stratification of 670 patients with advanced renal cell carcinoma. *J. Clin. Oncol.* **17**, 2530–2540.
9. Motzer, R.J., Bacik, J., Schwartz, L.H., et al. (2004) Prognostic factors for survival in previously treated patients with metastatic renal cell carcinoma. *J. Clin. Oncol.* **22,** 454–463.
10. Kaplan, E. and Meier, P. (1958) Nonparametric estimation from incomplete observations. *J. Am. Stat. Assoc.* **53,** 457.
11. Barney, J.D. and Churchill, E.J. (1939) Adenocarcinoma of the kidney with metastasis to the lung: cured by nephrectomy and lobectomy. *J. Urol*. **42,** 269–276.
12. Giuliani, L., Giberti, C., Martorana, G., and Rovida, S. (1990) Radical extensive surgery for renal cell carcinoma: long-term results and prognostic factors. *J. Urol.* **143,** 468–473; discussion 473–464.
13. Golimbu, M, Joshi, P., Sperber, A., et al. (1986) Renal cell carcinoma: survival and prognostic factors. *Urology* **27,** 291–301.
14. Maldazys, J.D. and deKernion, J.B. (1986) Prognostic factors in metastatic renal carcinoma. *J. Urol*. **136**, 376–379.
15. Neves, R.J., Zincke, H., and Taylor, W.F. (1988) Metastatic renal cell cancer and radical nephrectomy: identification of prognostic factors and patient survival. *J. Urol*. **139,** 1173–1176.
16. Pogrebniak, H.W., Haas, G., Linehan, W.M., et al. (1992) Renal cell carcinoma: resection of solitary and multiple metastases. *Ann. Thorac. Surg*. **54,** 33–38.
17. Kavolius, J.P., Mastorakos, D.P., Pavlovich, C., et al. (1998) Resection of metastatic renal cell carcinoma. *J. Clin. Oncol.* **16**, 2261–2266.
18. Wronski, M., Arbit, E., Russo, P., and Galicich, J.H. (1996) Surgical resection of brain metastases from renal cell carcinoma in 50 patients. *Urology* **47,** 187–193.
19. Kim, S.H., Brennan, M.F., Russo, P., et al. (1998) The role of surgery in the treatment of clinically isolated adrenal metastasis. *Cancer* **82,** 389–394.
20. Montie, J.E., Stewart, B.H., Straffon, R.A., et al. (1977) The role of adjunctive nephrectomy in patients with metastatic renal cell carcinoma. *J. Urol.* **117,** 272–275.
21. Walther, M.M., Yang, J.C., Pass, H.I., et al. (1997) Cytoreductive surgery before high dose interleukin-2 based therapy in patients with metastatic renal cell carcinoma. *J. Urol.* **158,** 1675–1678.
22. Rackley, R., Novick, A., Klein, E., et al. (1994) The impact of adjuvant nephrectomy on multimodality treatment of metastatic renal cell carcinoma. *J. Urol.* **152,** 1399–1403.
23. Sella, A., Swanson, D.A., Ro, J.Y., et al. (1993) Surgery following response to interferon-alpha-based therapy for residual renal cell carcinoma. *J. Urol.* **149**, 19–21.
24. Flanigan, R.C., Salmon, S.E., Blumenstein, B.A., et al. (2001) Nephrectomy followed by interferon alfa-2b compared with interferon alfa-2b alone for metastatic renal-cell cancer. *N. Engl. J. Med.* **345**, 1655–1659.
25. Mickisch, G.H., Garin, A., van Poppel, H., et al. (2001) Radical nephrectomy plus interferon-alfa-based immunotherapy compared with interferon alfa alone in metastatic renal-cell carcinoma: a randomized trial. *Lancet* **358**, 966–970.
26. Flanigan, R.C., Mickisch, G., Sylvester, R., et al. (2004) Cytoreductive nephrectomy in patients with metastatic renal cancer: a combined analysis. *J. Urol.* **171,** 1071–1076.

27. Finelli, A., Kaouk, J.M., Fergany, A.F., Abreu, S.C., Novick, A.C., Gill, I.S. (2004) Laparoscopic cytoreductive nephrectomy for metastatic renal cell carcinoma. *BJU Int.* **94,** 291–294.
28. Bromwich, E., Hendry, D., and Aitchison, M. (2002) Cytoreductive nephrectomy: is it a realistic option in patients with renal cancer? *BJU Int.* **89,** 523–525.
29. Melchail, T.M., Abbou-Jawade, R.M., Bournrhi, G., et al. (1999) Validation and extension of the Memorial Sloan Kettering prognostic factors model for survival in patients and previously untreated metastatic renal cell carcinoma. *J. Clin. Oncol.* **17,** 2530–2540.
30. Eggener, S.E., Yossepowitch, O., Pettus, J.A., Snyder, M.E., Motzer, R.J., and Russo, P. (2006) Renal cell carcinoma recurrence after nephrectomy for localized disease. Predicting survival from the time of recurrence. *J. Clin. Oncol.* **24**, 3101–3106.
31. Kim, W.Y. and Kaelin, W.G. (2004) Role of VHL gene mutation in human cancer. *J. Clin. Oncol.* **22**, 4991–5004.
32. Drevs, J., Hofmann, I., Hugenschmidt, H., et al. (2000) Effects of PTK787/222584, a specific inhibitor of vascular endothelial growth factor receptor tyrosine kinases, on primary tumor, metastasis, vessel density, and blood flow in a murine renal cell carcinoma model. *Cancer Res.* **60,** 4819–4824.
33. Larkin, J.M. and Eisen, T. (2006) Kinase inhibitors in the treatment of renal cell carcinoma. *Crit. Rev.Oncol. Hematol.* [epub ahead of print]
34. Ahmad, T. and Eisen, T. (2004) Kinase inhibition with BAY 43-9006 in renal cell carcinoma. *Clin. Cancer Res.* **19,** 6388s–6392s.
35. Yang, J.C., Haworth, L., Sherry, R.M., et al. (2003) A randomized trial of bevacizumab, an anti-vascular endothelial growth antibody for metastatic renal cancer. *N. Engl. J. Med.* **349,** 427–434.
36. Sakamoto, K.M. (2004) SU-11248 Sugen. *Curr. Opin. Investig. Drugs* **5,** 1329–1339.
37. Dutcher, J.P. (2004) Mammalian target of rapamycin inhibition. *Clin. Cancer Res.* **10,** 6382–6387S.
38. Ratain, M.J., Eisen, T., Stadler, W.M., et al. (2006) Phase 2 placebo-controlled randomized discontinuation trial of sorafenib in patients with metastatic renal cell carcinoma. *J. Clin. Oncol*. **24,** 2505–2512.
39. Motzer, R.J., Michaelson, M.D., Redman, B.G., et al. (2006) Activity of SU11248, a multi targeted inhibitor of vascular endothelial growth factor receptor and platelet-derived growth factor receptor, in patients with metastatic renal cell carcinoma. *J. Clin. Oncol.* **24,** 16–24.
40. Motzer, R.J., Rini, B.J., Bukowski, R.M., et al. (2006) Sunitinib in patients with metastatic renal cell carcinoma. *JAMA* **295,** 2516–2524.
